# Can Occupational Therapy Address the Occupational Implications of Hoarding?

**DOI:** 10.1155/2019/5347403

**Published:** 2019-03-04

**Authors:** Cathy Clarke

**Affiliations:** School of Allied Health Professions and Midwifery, Faculty of Health Studies, University of Bradford, Richmond Road, Bradford BD7 1DP, UK

## Abstract

Hoarding is often described as a medical disorder, defined by a persistent difficulty in discarding possessions and associated high levels of emotional distress when forced to part with these. This article will discuss how having a different view of hoarding, seeing hoarding as a daily occupation which provides value, purpose, and meaning and with a relationship to self-identity and life purpose, could offer alternate interventions to support an individual who hoards. The article will consider the components of hoarding activity and how these relate to health and wellbeing and doing, being, belonging, and becoming as understood by occupational therapists. The article will consider what occupational therapy, a profession which considers a person's daily occupations, the things that occupy their time and which give meaning to their existence, could offer as an alternative to current hoarding interventions. Proposals for occupational therapy interventions will be suggested which would support occupational choice, support engagement in activities which have more positive outcomes on a person's health, and seek to address barriers which limit engagement and occupational performance in activities within the person's home environment.

## 1. Introduction

Occupational therapy aims to support a person's health and wellbeing, incorporating the fundamental values and beliefs of the profession, in using meaningful and purposeful occupation as a vehicle to promote and sustain positive health and lifestyle [1]. However, whilst it has recently been acknowledged that some occupations can be detrimental to a person's health, safety, and wellbeing [2], there is a lack of occupational therapy literature to explore such occupations. In addition, there has been a lack of discussion on the challenges for occupational therapists in working with those whose occupations cause detrimental impacts on health and daily life and in exploring ways to work with these people and populations without using reductionist approaches to stop what are deemed nonsanctioned occupations [3, 4]. The term nonsanctioned occupations as described by Kiepek et al. [4] refers to occupations which tend to be viewed as unhealthy, undesirable, unacceptable, and/or inappropriate and are often connected to historical and cultural perceptions.

Evidence shows that hoarding is an activity which can have detrimental effects on a person's health and wellbeing through difficulties in not being able to use areas of the home as intended, restricted engagement in home activities, and increased risks of falls, leading to poor physical health and psychological wellbeing [5]. It is also evidenced that hoarding is seen as an unhealthy and undesirable activity by the public, both in relation to the occupation itself and the impact on a person's home because of the collected items [6].

The definition of hoarding, as described by the American Psychiatric Association (APA) [7], refers to the persistent difficulty in discarding or parting with possessions due to a perceived need to save and retain items, as well as a need to avoid the negative emotions experienced in discarding these. The definition provided by the APA [7] however does not refer to hoarding as an activity but has more of an emphasis on the difficulties the person experiences in parting with their possessions. However, hoarding can be seen as an activity which encompasses a series of tasks which relate to the collection of and saving of meaningful and valuable items. It includes daily habits and routines that structure these tasks, and as a person's daily occupation which fills the person's waking hours, it encompasses roles and has purpose. Thus, hoarding encompasses a number of differing activities that relate to the acquisition and retention of objects and can also be viewed as an occupation which has purpose and constructs the meaning use of a person's time and home environment [8]. However, the understanding of hoarding as an occupation also requires appreciation that this is an activity that is not necessarily socially acceptable to the public, and may infringe on their rights, but still has meaning and purpose to the individual who engages in hoarding activity. This allows understanding of “dark” or “nonsanctioned” occupations, appreciating that people will engage in occupations which challenge the sociocultural and health constructs which are more dominant in the population [9], but that these are still relevant areas for occupational therapists to consider to ensure practice upholds the occupational rights of all people and populations [10].

Occupational therapy considers a person's daily occupations, the things a person does to occupy their time and which gives meaning to their existence; as such, hoarding viewed as a purposeful daily activity is of interest to the profession. However, to date, very few studies have been conducted which explore the role of occupational therapy in supporting those who engage in hoarding activity.

This paper will discuss how the beliefs of occupational therapy, the value and purpose of daily occupation, can be used with occupational science to reframe understanding about hoarding and from this propose how occupational therapy could offer alternative approaches and interventions to what are currently provided to support those who hoard.

## 2. Method

A review of literature was conducted using key words, with combinations of terms to ensure relevant literature was considered. A systematic process was used to review, interpret, question, and further review literature in an iterative and incremental way; the method did not follow a systematic review process as this was perceived as potentially limiting the exploration of the literature fully. The process included the examination and critical consideration of existing knowledge relating to hoarding; this was then considered in relation to the theoretical principles and the practice skills of occupational therapy, in order to propose practice frameworks which could support the emerging role of occupational therapists in working with those who hoard.

Table 1 provides details of search terms and inclusion criteria.

Date limits were applied to exclude hoarding studies conducted prior to 2000; this enabled consideration of more recent literature pertaining to treatment of hoarding. No date restrictions were applied when reviewing literature on occupational therapy and hoarding. Literature which included “children and hoarding” was excluded as the author wished to explore the specific aspects of hoarding related to adults for this review, and thus, search criteria focused on adults 19 years and over. Literature was also excluded which considered specific types of hoarding, such as the collection and retaining of animals, shopping, or retaining very specific objects. Literature which indicated that participants included in studies had additional diagnosed health conditions, e.g., dementia, attention deficit disorder, or behaviours associated with mental illness or mental incapacity, was also excluded as these did not relate to the DSM categorisation of hoarding.

Literature was sourced from databases including PubMed, Medline, ScienceDirect, and CINAHL. In addition, hand searching of journals and textbooks and sourcing of the internet for any additional materials also provided further materials. Reference sections of sourced published articles were also examined when deemed relevant to the literature review. As the process was not conducted as a systematic review, the process considered a wide range of academic fields and did not focus on any specific disciplines. However, on reflection, this leads to a limitation in the range of literature as the majority of literature, sourced from the databases, indicated medicalised and pathological views of hoarding.

From the initial search, it became clear that the search term “hoarding” did not provide much literature compared to when the term “hoarding disorder” was used. This is of interest as it potentially indicates that hoarding is more often viewed as a medical disorder rather than a way of life and daily occupation for some people. Whilst previous literature is of interest, this paper seeks to explore hoarding as an occupation, and thus, there is an ongoing intentional discourse presented throughout this paper, between the evidence generated from previous literature which has mainly presented hoarding as a disorder in need of remediation, contrasted with discussions of hoarding as a daily occupation that is a chosen activity seen to benefit the person's life. The lack of literature, discussing hoarding as a daily occupation, was evident from results from database searches, when no literature was produced using the search terms “hoarding and occupational therapy/occupational science” or “occupational therapy and hoarding disorder,” which suggests that there is a lack of research in this area. Any pertinent literature on occupational therapy and hoarding was retrieved from general internet searches and specific exploration of occupational therapy journals, but the evidence of occupational therapists working with hoarders was very limited, and there was no literature evidencing research on the practice of occupational therapists with this population.

Legislation related to hoarding within the UK was explored in order to understand the relevance in relation to the management of hoarding under public health acts, and grey literature, such as working papers on interventions to address hoarding, policy statements on hoarding, and information produced by the Department of Health, sourced from the internet, were also considered.

## 3. Literature Review

### 3.1. Professional Disciplines and Previous Studies

From the literature review, it was evident that the main body of hoarding research had been conducted by professionals with psychology and/or psychiatry backgrounds, with psychologists providing the majority of interventions, using cognitive behaviour therapy models to underpin approaches [11]. Thus, interventions were underpinned by medical models/frameworks, with very little acknowledgment of the limitations in such approaches. Whilst the DSM diagnosis usefully separates hoarding as a condition in its own right, it also has the potential to medicalise the problem; the lack of acknowledgment, of the psychological aspects of conditions when classified through the DSM classification, has been noted by Kinderman et al. [12] and proposed as leading to a lack of understanding of health conditions and in providing appropriate interventions. This can be seen in the approach to address hoarding when the adoption of a medical perspective appears to govern a focus on problem-solving interventions that sees the removal of the collected items, with some additional cognitive behaviour therapy to remediate the person's thinking and behaviour in order to stop them in the act of hoarding, as the solution. However, such approaches seem to fail in acknowledgment that hoarding constructs daily life activities and that these will not be simply stopped by the removal of collected items from the home. In addition, whilst these approaches, of removing collected items, are proposed to improve the person's quality of life, it is acknowledged that this fails to acknowledge the psychological distress the person experiences [13].

### 3.2. Participants Included in Previous Studies

Studies conducted prior to 2013 had viewed hoarding within the category of obsessive compulsive disorders (OCD), and thus, the selection of participants within these studies had not just included people who hoard but had also encompassed those with OCD disorders. However, Frost et al. [14] reported that a large percentage of people who engaged in hoarding activity did not experience obsessive compulsive disorders, but despite this research, studies prior to 2013 did not differentiate participants, and thus, people who engaged in hoarding were included with those who were diagnosed as having OCD. As such, Bloch et al. [15] and Ong et al. [16] stated that outcomes from studies prior to 2013, pertaining to causes and treatments of hoarding, were subsequently flawed. However, Burns [17] referred to the difficulties in recruiting participants into hoarding studies due to individuals feeling shame and embarrassment because of their behaviour and the physical states of their home environments, with additional fear that becoming visible as a person who hoards would result in forced decluttering. Thus, the actual number of people who hoard compared to the number of people who had OCD included in studies may have been very low. Actual evidence on the number of people who hoard is also difficult to estimate, with variances from 2.3% in the UK [18] to 3.7% in the United States [19] and 4.6% in Germany [20]. However, as these studies were conducted prior to 2013, data may again be flawed, with participants in the studies possibly including those with OCD and not necessarily just those who hoard.

The majority of hoarding studies were conducted in more affluent Western populations where the acquisition of material objects has more favour and is related to social status and roles [21, 22]. Whilst such social perspectives may have influenced the data from studies conducted on hoarding, this was not always acknowledged.

Rules for expected human behaviours are generally underpinned by social, political, economic, and cultural policies, which mean that hoarding behaviour may be classified according to the rules of each society [23]. This results in differing public and health provisions to address hoarding behaviour, with Chia et al. [24] and Papadopoulos et al. [25] noting the need for more research in order to understand the impact of culture in relation to hoarding and for more consideration of appropriate public and health responses relative to sociocultural determinants. This would seem to indicate a need to explore interventions which focused on public health approaches rather than treating hoarding as a medical disorder; however, despite this, there was a lack of literature which explored a more sociological approach, to supporting those who hoard.

### 3.3. Hoarding and Occupational Therapy

A review of occupational therapy literature yielded few studies about the profession's role in working with people who hoard. Spear [26] suggested that occupational therapy could consider the roles, routines, and habits that were part of hoarding behaviour and the relationship of these to occupational performance, utilising this knowledge to develop relevant occupation-focused approaches. In addition, an exploratory study conducted in 2017 [27] proposed that there was a role for occupational therapy to address hoarding but noted the absence of any specific evidence for interventions. The authors concluded that whilst there was evidence to indicate the impact of hoarding on occupational functioning, there was no published research regarding the contribution that occupational therapy or occupational science could make to support those who engaged in hoarding as a chosen daily activity.

### 3.4. Assessment Tools Used in Hoarding Studies

A number of assessment and intervention tools have been developed to address hoarding [11, 28]; however, many of these have been developed by psychologists. Some of these would seem to have relevance to the focus of occupational therapy, e.g., Activities of Daily Living Scale [11], in considering daily activities and occupations; however, there is a need to ascertain their value when used within occupational therapy interventions. It should also be considered whether these assessment tools adopt a medicalised view of hoarding.

## 4. What Is Hoarding?

### 4.1. Classification of Hoarding

Prior to 2013, hoarding was classified within the category of obsessive compulsive disorders; however, a new classification in 2013 defined hoarding as a discrete and specific disorder in its own right (American Psychiatric Associations (APA)). This more recent definition refers to hoarding as persistent difficulty in discarding or parting with possessions due to a perceived need to save items and objects, as well as a need to avoid feelings of distress in discarding them (DSM-5, [7]).

The difficulty, in not discarding collected items in hoarding, means that the accumulation of possessions becomes excessive, to the point where space, safety, and the intended use of the home living environment are compromised [7]. In the DSM-5 definition of hoarding, the specific need to collect items is not attributed to any other mental health conditions such as dementia, brain injury, or cerebrovascular disease.

The DSM-5 [7] definition of obsessive compulsive disorders refers to such conditions as primarily being anxiety disorders, with the individual experiencing repetitive thoughts that lead them to engage in specific actions and behaviours. The anxiety symptoms relate to the perceived consequences of not undertaking set actions and behaviours, as well as anxiety as to what would happen if these were not conducted [29]. In hoarding, the anxiety arises due to the fear of being parted from possessions rather than anxiety related to the actual activities of hoarding and collecting, with these activities experienced as pleasant and meaningful by the person [30]. There can be seen therefore a difference between the definitions of anxiety experienced by someone who hoards compared to the anxiety experienced by someone with OCD, and this should be acknowledged when considering interventions, as treatments to address OCD may not be effective for those who hoard [15].

### 4.2. Collecting and Retaining Items

Collecting and retaining items are normal human activities with people intentionally seeking and collecting meaningful required resources and objects [31], with these having intrinsic, instrumental, and sentimental attachments to the person who has intentionally sourced these. Thus, the activity of collecting items does not necessarily indicate hoarding behaviour. The word “clutter,” referred to by authors in previous literature, has been used to describe unwanted/unneeded items; however, this is in contrast to descriptions by the person who hoards, who states these items are valuable and meaningful and refutes suggestions that the items are unnecessary or “clutter” [32]. The meaning of the word “clutter” does seem to relate to the stance of the person using it, as it would seem previous studies, conducted by psychologists or psychiatrists, refer to “clutter” as unwanted or needed items, and legislation, such as the Environmental Protection Act [33] and the Public Health Act [34], use the words “litter” and “clutter” to describe items which are deemed to be adversely affecting a person's property and/or external environment and as such should be removed. Thus, the use of “clutter” or “litter” would seem to indicate a viewpoint that the items are not of worth and therefore not needed. It is acknowledged that there are differing viewpoints of phenomenon [35]; however, the perception of the value, or not, of the collected items in hoarding appears to have direct links to the approaches used in response to those who hoard and that these perceptions are also aligned to the sociocultural stance of the communities in which the person who hoards resides. However, whilst the majority of previous studies on hoarding have framed the collected items as “clutter,” this paper seeks to offer a differing perception, based on occupational perspectives, which proposes that collected items have meanings connected to the activity of hoarding and thus are not unnecessary or unwanted by the person.

Studies show that collected items become increasingly excessive in amount, causing detrimental effects limiting the space in the home [36] and in the person's ability to use areas of the home as originally intended [11]. However, the person who hoards does not experience distress because of these situations [37] and does not seek help or assistance from friends, family, or public services as they do not believe that the removal of items will improve their quality of life or their abilities to perform tasks [16, 17, 38]. Saxena et al. [39] note that hoarders report they achieve a quality of life associated with the attainment of hoarding goals and from feeling connected to items that they have collected. They associate meaning and purpose to the hoarding activity and have strong psychological positive links between retained possessions, wellbeing, and meaningful existence.

### 4.3. The Impact of Hoarding

Whilst it has been shown that the accumulated items retained by the person can prevent them from engaging in activities, such as sleeping in bed, sitting in living rooms, or cooking in kitchens [37], the presence of personally meaningful items in any individual's home is noted to have a positive effect on self-identity [40, 41]. In addition, if home is seen as a place where meaningful and purposeful activities can occur [41] and personal possessions within this space provide a sense of self-identity [40], Belk et al. [21] report that psychological wellbeing is enhanced. Thus, whilst Manzo [42] and Roster et al. [43] report that there is an adverse impact on the psychological health of the person who hoards because of excess items cluttering the home, there is an argument that these collected items create a therapeutic refuge within the home [44] and enable the individual to use objects in purposeful and meaningful activities [45].

The differing of opinions between the person who hoards who view the items as “clutter” as opposed to the person who hoards seeing these as having value and purpose can create friction which can cause family relationships to break down and for the person who hoards to become defensive towards offers of help from health and social care services [17].

Within the United Kingdom, the implications of hoarding, such as accumulated items adversely impacting on the home environment and the health and safety of the individual or adversely affecting neighbours' health and wellbeing because of the unhygienic situation of the person's home and the immediate vicinity, can invoke responses from environmental health services, underpinned by legislative powers [33, 34]. The Public Health Act [34] underpins actions to clear properties and remove items, in or outside of a property, if these items are deemed prejudicial to health. However, as previously noted, the person who hoards views collected items as beneficial to their health and of value to others [46] and thus is in contrast to the legislative viewpoint of collected items being hazardous to health. Removal of items, commonly referred to as decluttering, has financial costs which are paid by the local council and the person themselves [17], which whilst instigated by others requires the individual themselves to pay for removal of items they wish to keep, which brings concerns from an occupational justice perspective.

The Environmental Protection Act [33] is instigated when the state of premises is prejudicial to health or a nuisance to the environment, and this is often implemented when the state of a person's home, through hoarding, has a detrimental effect on neighbours or the neighbourhood. Such decluttering responses were noted in studies conducted by Brakoulias and Milicevic [47] and Gordon et al. [48], when neighbours raised complaints about the impact of collected items on sanitation issues within the neighbourhood. Complaints by neighbours and friction between family and friends who are unable to comprehend the reasons for the individual engaging in hoarding activity and refusal to remove items from the home can lead to the person becoming marginalised and socially rejected [49, 50]. This has also been acknowledged by Burns [17], who reported significant social stigma experienced by people who hoard and that this also extended to the people who resided with them, such as family members. Pertusa et al. [51] stated that this stigma and fear prevented people from accessing health and other support services, ultimately creating a hidden marginalised population. Thus, legislative responses appear to protect the public from poor hygiene and sanitation aspects which may arise from the environment of someone who hoards and seem to follow what is deemed acceptable behaviour and ways of living with the dominant sociocultural expectations of the community; however, they fail to acknowledge the occupational rights of people in choosing to live in ways that may be in contrast to the dominant society, with this acknowledged by Kiepek et al. [4] as resulting in the marginalisation of people because their behaviour and actions are seen as “deviant.”

Economic costs associated with hoarding include work absenteeism [52] and costs for provision of enhanced medical care. The later aspects can include the provision of mental health interventions for conditions associated with hoarding such as depression and also medical treatments for chronic and severe medical problems such as diabetes and cardiovascular conditions, common in people who hoard [53]. The impact of accumulated items in the home environment can also increase the risk of falls [54] and bring about self-neglect because of inabilities to engage in self-care if access to the bathroom is prevented or through not being able to make meals if the kitchen is inaccessible or unhygienic due to excessive items [37, 55].

Fleury et al. [6] noted the socioeconomic costs associated with hoarding, with Brown and Pain [30] specifically citing costs arising from responses by Fire and Police services. In these situations, services are often required to visit hoarding properties to provide interventions to prevent risks of fires and falls due to excessive items being flammable and in restricting safe mobilisation. Kim et al. [56] also noted financial costs when children were removed from homes due to accumulated items affecting their safety and welfare, requiring input from Child Protection Services.

It is interesting to note that the impact of hoarding has mainly been considered by people external to the hoarder and this can result in hoarding being perceived as a nonsanctioned occupation which needs to be changed. From the literature review, very few studies considered the perspective of the hoarder and what support/assistance they required which has possibly led to services being “done” to the hoarder rather than “with” the person and in meeting their choices.

## 5. Why Do People Hoard?

Seaman et al. [57] suggested that hoarding develops because of distorted cognitive beliefs. According to Seaman et al. [57], these included fear that harm would come to the individual, or others, if objects are not retained, that memories would be lost or emotional and physical discomfort experienced if objects were discarded. Saxena et al. [58] and Grisham et al. [59] suggested that hoarding was perpetuated because of poor cognitive skills and reduced attention, causing difficulty in completion of spatial tasks, problem-solving, and decision-making. These deficits make it difficult for the person to focus, organise, sort, and categorise objects, leading to excessive accumulation of objects and inability to manage these. Kellett et al. [32] proposed that hoarding activities become engrained in daily life, structuring thinking and actions to the point where the individual feels a psychological addiction to continue in this way, with Kellett et al. [32] referring to this as being psychologically trapped by the actions of hoarding. It could therefore be proposed that hoarding becomes an addiction generated by the underlying intrinsic motivators to hoard and retain objects, with any attempts to refrain from this behaviour resulting in extreme psychological distress.

## 6. Current Treatments to Address Hoarding

From the literature review, it was apparent that cognitive behaviour therapy (CBT) had been the most dominant intervention to address hoarding [47, 60], with this seeking to stop the person completing hoarding activity by changing their thoughts and patterns of behaviour. However, Brakoulias and Milicevic [47] reviewed that only 20% of cases commonly received CBT, and whilst research indicated some slight beneficial effects, it also evidenced poor treatment outcomes and poor compliance from the individual, with reduced motivation to change behaviours [53]. Thus, previous interventions, which aim to readdress thinking and behaviours in people who hoard, appear to not be strongly substantiated. When decluttering is included as part of the intervention, Rodriguez et al. [61] noted that the individual also experienced increased levels of emotional distress. The increased levels of emotional distress that occur from forcible removing possessions that have intrinsic, instrumental, and sentimental connections to the person and which represent parts of themselves through self-identity [13] are understandable, yet may not be considered within a decluttering intervention. In addition, the outcome of the decluttering process results in a property that no longer represents the individual and their definition of home, with all items meaningful to them removed [61]. Home, according to Werner et al. [62], is a place where people experience safety, are able to engage in home-making activities which are productive or relaxing, and that have family and cultural connection, with home being a place where the person and their home environment cannot be defined separately and are mutually defining. Thus, for most people, home is a place which has meaning and enables them to engage in chosen and purposeful activities which reflect aspects of themselves. Whilst decluttering is seen by those who do not hoard as a process of removing items which are hazardous to the individual and limiting the use of the home environment, the author proposes that the process removes objects which directly relate to the person, disconnecting them from objects which define them. Thus, whilst previous studies have discussed the psychological distress associated with the decluttering process [60], it seems understanding from this has been based on the loss of possessions only and not the loss of connection and meaning of home; it can be understood therefore why this approach is opposed by those who hoard [6]. The overemphasis on decluttering homes and discarding possessions may lead to hostility which further deters individuals from accepting family and professional help. In addition, literature indicates that decluttering does not address the underlying causes of why someone hoards, and thus, hoarding behaviour perpetuates [6].

Behavioural and pharmacological treatments show some beneficial effects for obsessive compulsive disorders, with antidepressants commonly prescribed, but these approaches are shown to not be as effective when used to treat hoarding [15], and these approaches seem to fail to acknowledge the differences between OCD and hoarding.

The 2013 classification of hoarding as a unique condition gives possibilities for people who hoard to seek financial support through their health insurance for differing treatment approaches [44]. Proposals for differing approaches are suggested by Steketee and Frost [63], who recommend a better understanding of the individual's thoughts related to the acquisition and disposal of objects and a focus on interventions which encompass shared agreements and goals which aim to change the pattern of hoarding activity. However, there still remains an absence of health or social care professional guidelines for the management and treatment of hoarding [37]. Recommendations from Ayers at al. [53] and Brown and Pain [30] suggest that the most effective approaches should be ones that incorporate relationships of trust and client centeredness between services and the person who hoards and that a multiagency/multidisciplinary approach to achieve a common collaborative goal is ideal [64].

A task force response established to consider approaches to hoarding in America in 2012 acknowledged that occupational therapy could provide value within collaborative responses; however, at the time, no occupational therapists were employed within the service [65].

## 7. Viewing Hoarding from an Occupational Perspective

Occupational therapy is concerned with supporting a person's health and wellbeing through engagement in meaningful and purposeful occupations [1]. However, whilst it is important to support individuals to engage and perform in chosen activities and for individuals to have opportunity to engage in meaningful daily occupation, the profession recognises that some chosen activities can have detrimental effects on a person's health, wellbeing, and safety [2]. Evidence shows that hoarding is an activity which can have detrimental effects on a person's health and wellbeing, with collected items adversely impacting on the intended use of the home, the person's safety in the home, and their abilities to complete daily activities such as self-care [5]. In addition, the collection of objects can also lead to other occupational restrictions such as social marginalisation from family and friends [55], with people choosing not to visit the person's home, and social stigmatisation due to being identified as someone who hoards [50] which can cause further social isolation.

Occupational therapy quite often seeks to challenge the medical model, opposing the use of bottom-up reductionist approaches which focus on remediating functional problems in the individual; however, if the culturally predominate stance of the organisation uses this framework to underpin models of treatment, an occupational therapist may find it difficult to challenge this [66, 67]. Within hoarding interventions, this could result in the occupational therapist being involved in the removal of excessive items in someone's home as thus would align with a reductionist approach, viewing decluttering as the way to stop someone hoarding and preventing them from reengaging in this in the future. However, using top-down interventions, which look for ways to enhance performance in people [68, 69], as frameworks to underpin work with those who hoard, could allow occupational therapists to support individuals to reengage in activities in their home which may have been prevented due to excessive items limiting space in the home or to choose more health-sustaining occupations to replace hoarding activity.

### 7.1. Hoarding as an Activity

Humans are occupational beings [70], individuals who purposefully engage in activities which not only support biological needs but which also provide meaning and structure to daily life. These activities are structured into roles, routines, and habits, and it can be helpful to consider these within the framework proposed by Wilcock [70], which considers how the aspects of “doing, being, belonging, and becoming” relate to motivation for doing activities.

#### 7.1.1. The “Doing” within Hoarding

Understanding doing, as a component of meaningful activity, is a central element of occupational therapy [71]. Within the context of hoarding, the occupational element of doing includes the acquisition of and sorting of objects. It requires the person to use physical and psychological skills and includes roles and routines nested in behaviours which structure the person's day [72]. However, whilst hoarding is perceived as a useful and valuable activity by the individual themselves, others, such as family, friends, and public health and care services, are unable to perceive the same purpose and value, leading to emotional friction.

Being engaged in chosen and meaningful activities is seen as beneficial to health [44], and for the person who hoards, there is a sense of value in the activity, with the acquired objects providing sentimental and/or instrumental purpose [54]. However, hoarding adversely affects a person's health because of the impact of collected items in the home environment and abilities of the person to engage safely in activities within the home. In addition, hoarding activity can have additional risks which can occur through the act of collecting some unsanitary or potential harmful items such as bodily fluids [37] or out of date foods or due to risks associated with the environments the person visits to collect items [6]. Risks from exposure to extreme weather conditions during the collection process such as cold or heat can also have detrimental effects on the person's health and wellbeing. The profession of occupational therapy is concerned with promoting the health and wellbeing of people through engagement and performance in meaningful activities [1, 73]. The profession also supports concepts of occupational rights and aims to ensure that a person is able to engage in meaningful activities that contribute positively to their wellbeing and the wellbeing of their communities [74]. There is therefore the opportunity, from this professional perspective, to acknowledge that hoarding provides intrinsic value to the individual but to also acknowledge that engagement in hoarding does not positively support the person's physical health and wellbeing and that it can also adversely affect the wellbeing of others due to the sanitation impact on neighbourhoods. There is therefore an opportunity for occupational therapy to engage with a person who hoards to support them to engage in more health-sustaining activities and to support their roles and acceptance within communities.

Functional skills can be lost if the person is physically unable to engage in some activities because of restricted access to some areas of the house, such as the kitchen and bathroom, due to excess items blocking access. This can create occupational dysfunction with individuals loosing physical and/or cognitive skills to complete some tasks due to the lack of engagement, resulting in inabilities to accomplish tasks they were previously able to do [8].

If access to the bathroom is restricted, the individual is unable to engage in self-care activities which can have adverse impact on their wellbeing. The lack of clean and safe and working areas in the kitchen for food preparation can also increase risks to the person's health and reduce their overall nutritional intake.

#### 7.1.2. The “Being” within Hoarding

Self-identity develops from having a sense of purpose and encompasses roles and routines and characteristics which are unique to a person [75]. Generally, people are able to engage in a balance of activities and tasks which encompass differing roles, and they are able to simultaneously complete these differing roles and associated tasks. This means that a role such as worker, mother, father, or friend or a particular task does not specifically define them. However, the person who hoards can spend a significant amount of time acquiring objects, incorporating specific routines in this activity, and this is to the detriment of other daily activities. Their daily activities are focused on hoarding and this is likely to lead to the person being defined as a “hoarder,” as this as their only role. Due to this focus on hoarding activity, the person may not be able to maintain a balance with other required activities and occupations. This can be referred to as having occupational imbalance [76] and occurs when the person is overwhelmed by one sole activity, with this adversely affecting their physical and psychological health.

The concept of “being” as understood by occupational therapy also relates to the concepts of being human, with this defined by Wilcock [77] as an occupational being who has a lived existence. Within this definition however is an expectation that as a human being, individuals are aware of their existence alongside other human beings and thus are able to consciously consider their behaviours and actions beyond their own immediate needs. However, within hoarding, this is not necessarily the case, as the need to engage in hoarding activity takes precedence over all other life aspects and awareness of others, thus indicating some lack of insight into the severity and impact of hoarding activity. In furthering this concept to consider cultural aspects of “being” to hoarding, there is acknowledgment that the majority of hoarding studies have been with Western populations, where an individualistic sense of self rather than collective view of self is promoted, with the acquisition of material objects having more influence in determining social status.

#### 7.1.3. “Belonging” within Hoarding

Whilst Wilcock [77] relates belonging as the connectedness of people to each other, which includes social interactions, friendship, and a sense of inclusion, this aspect is more challenging for people who hoard. The activities of hoarding can prevent the individual engaging in social occupations. The excessive amount of collected items can prevent activities occurring inside the home and thus prevent connection to others through social activities; this may also be exacerbated due to the social stigma associated with hoarding behaviour. The accumulation of objects within the home may lead to family and friends withdrawing contact, which can increase the likelihood of the person becoming socially isolated. The impact of entrapment experienced by those who hoard, described by Grisham et al. [59] as the consequence of the individual being physically trapped in their home due to excessive items and psychologically trapped in a role defined according to their behaviour, also contributes to social isolation.

Molineux and Baptiste [71] refer to the status of “belonging” as being connected to principles of giving and contributing to others, which would be an accepted concept by someone who hoards, as their beliefs underpin their actions around sustainability of resources being beneficial to others and that retained items will be of value to others in the future [46].

#### 7.1.4. “Becoming” within Hoarding

There would appear to be an association with hoarding activity and aspects of becoming, with collected items relating to the individual's future goals and aspirations and having connection to their beliefs of who they will “become.” The motivation and reason for engagement in any activity are unique to a person [75, 78] and relate to their immediate reason for engaging in activities, as well as their future goals. Within hoarding, the unique reason why a person engages in hoarding activity, the reason they seek and collect objects, is related to their motivation of doing the activity, and this can be for a number of reasons. It can be because they believe the object will be useful or valuable in the future, or there is a sentimental value in keeping the object. Hoarding can also develop from an initial hobby of collecting specific items, which then develops into an all-consuming activity, which replaces time spent engaged in other activities and results in collected items overwhelming space inside the home. In this situation, Cutchin et al. [79] note that the initial goals and aspirations for collecting items within the activity of a hobby become distorted, causing the potential difficulties associated with hoarding.

The opportunity for an individual to engage in meaningful occupations, to meet their needs and develop their potential, is an occupational right, acknowledged by Durocher et al. [80]. Wilcock [77] and Stadnyk et al. [81] refer to principles of occupational justice, in that people have the right to engage in meaningful and purposeful occupations, with Wilcock stating that in an occupationally just world individuals would be supported to enhance their wellbeing by doing what is most meaningful to them, and their families, and communities. The actions of decluttering and further legislative rules under the Public Health Act [34] applied to prevent a person reengaging in the occupation of hoarding can be described as acts which prevent occupational justice, in that they prevent the person doing what is meaningful to them. This can also be described as the person experiencing occupational deprivation, with Wilcock [77] explaining that this can occur when environmental, social, or cultural issues dictate the required or expected ways of being and behaving. Forced decluttering does not seem to recognise that retained items connect with the individual's self-identity, meaning of home, and aspirations and goals of the individual and therefore do not seem to align to principles of an occupationally just world. The process of decluttering can cause loss of identity and loss of the psychological meaning of home.

## 8. The Potential for Occupational Therapy in Working with Those Who Hoard?

Viewing hoarding as an activity which structures daily life and which has purpose and meaning to an individual can enable occupational therapists to consider alternative interventions to address hoarding to those that have been previously implemented. Using an occupational understanding of the world, as purported by Townsend and Polatajko [73], can support approaches and treatment interventions which accept that an individual's actions are meaningful and that they provide motivation for the activities they choose to perform. This is a useful paradigm to use when considering hoarding, as it enables a position of understanding of hoarding being a meaningful occupation to an individual and that it structures their roles and routines.

As health care professionals, occupational therapists aim to enhance peoples' health and wellbeing; their practice adheres to principles of respect for client autonomy and prevention of harm, and they offer interventions which support the capacity of individuals to exercise choice [82]. Therefore, acknowledging that evidence shows that forced decluttering causes intense levels of emotional distress [61], occupational therapists would not want to engage in such actions as these would contravene their professional codes of conduct. In addition, further evidence notes that forced decluttering does not have good long-term outcomes with high rates of recidivism for the person retuning to hoarding activity [65]. Thus, there is an opportunity to consider differing interventions which may have better long-term outcomes.

It is necessary however to acknowledge that whilst the profession aims to uphold aspects of occupational justice in context with moral, ethical, and health aspects in situations when a person's health, wellbeing, and safety are compromised to a point of serious concern because of hoarding, legislative actions by the Environmental Protection Act [33] and the Public Health Act [34] to forcibly remove items that are prejudicial to the person's health and wellbeing may override occupational rights [80].

Occupational therapy would acknowledge that the person who hoards is intrinsically driven to pursue their goals of collecting and retaining items as these connect with their concepts of “doing,” “being,” “becoming,” and “belonging.” Interventions that prevent the person from engaging in hoarding cause feelings of loss and grief, related to not only loss of possessions which would occur through a decluttering approach but also loss of purpose of activity and loss of a reason for existing and being if the person was prevented from engaging in hoarding activity. Occupational therapy interventions would support the individual to find alternative activities and occupations which could structure their day and give them back meaning and purpose. However, the aim would ensure that the new activities and occupations would have less detrimental impact on the person's health and wellbeing compared to engagement in hoarding activities. Such an approach would acknowledge the “doing” and “being” aspects that were meaningful, and which connected the person to hoarding as an activity, but would seek to channel these underpinning desires into more health-sustaining and occupationally balanced ways of being and doing.

The promotion of engagement in differing activities beyond hoarding can be used to redevelop lost skills such as cooking or self-care to enhance occupational functioning and engagement in activities; these would also aim to promote wellbeing and better occupational balance. Skills unique to occupational therapy to support reengagement in activities, such as pacing, grading, and adaptation of the environment [83], can ensure the individual is successful in new activities and thus gain independence and self-achievement. Using a client-centered and partnership approach [84] can enable the individual to have some control in choosing alternative activities which have more health-sustaining outcomes. Supporting the development of additional activities which encompass new roles and routines that are not connected with hoarding can assist in the development of new identities, enabling the individual to be perceived by family and communities beyond the role of a “hoarder.” Such interventions can promote “being” and “becoming” through the development of life skills which enhance current occupational performance and attainment of future goals. Enhancing such new roles can also address the social stigma and social isolation by providing occupational opportunities to engage in social occupations in and out of the home environment. As hoarding may have prevented the individual “belonging” to the family, society, and community, leading to loss of family roles/rituals/routines and fragmentation of family identity; an occupational therapist can support the person to reconnect with such roles. This can address issues noted by Sampson [50], when family conflict may have increased due to feelings of hopelessness and frustration in family members, when the person continue to engage in hoarding activity to the detriment of all other aspects of life and relationships.

Whilst legislative actions may lead to forced decluttering and subsequent prevention of reengagement in hoarding activities [85], it can also create occupational alienation, whereby the person is forced to engage in other activities which they do not wish to do. In these situations, occupational therapy interventions can support the person to make decisions and give them some control in the process of decluttering and choice of future activities. It can allow the individual to repurpose some of the collected items into differing activities, possibly preventing a wholescale decluttering situation. Assisting the person to reframe collected objects within new occupations can provide insight in understanding the reasoning which underpinned the rationale for the collection of the objects and possibly support suggestions for alternative activities such as working with recycling organisations if aspects of sustainability underpinned hoarding beliefs. Retained objects often have links to memories, and thus, they have sentimental value [44]. Therefore, an approach which enables collected item to be either repurposed into other items which have meaning and value, such as using retained items of clothing and making into quilts, or saving items in alternative versions, such as paper photographs into digital form, can reduce the number of items in the home whilst respecting the value the items hold for the individual. Such approaches have been shown to be helpful in supporting individuals to repurpose collected items [44, 60]; however, such studies were conducted by psychologists whose aim was to minimise wholesale decluttering of collected items, and thus, there was not necessarily a focus on the meaning and value of retained items. There is merit therefore for occupational therapists to consider supporting individuals to engage in meaningful activities which can repurpose these collected items rather than interventions which are implemented to prevent wholescale decluttering.

Occupational therapists can enable individuals to develop life skills [86, 87], and as previous hoarding studies suggest that people who hoard may experience problems with cognition and problem solving [57, 58], the development of life skills to enhance occupational performance has possible merit. The development of such skills may also prevent the continued acquisition of objects as the person can begin to make better decisions about what to collect and enhance their problem-solving skills in organising, sorting, and categorising objects more effectively. This may also address the problems identified by Fleury et al. [6] that people who hoard often come back to the attention of public services after decluttering as they have not developed skills to better manage items if they reengage in hoarding activity.

Addressing physical barriers to engagement and performance in activities is a core skill of occupational therapy [1] and can be used in hoarding situations to address the impact of restricted environment and enhance the opportunities to engage and perform in differing occupations. This approach could be used as a motivational lever in enabling the individual to see the benefits of removing excessive items in the home in order to engage in other meaningful activities. Such motivational approaches are noted as successful when working with people who struggle to take the first step in addressing problems associated with addictive behaviours [88], and within hoarding, there is an addiction to perpetuating collection of objects.

Occupational therapy approaches would not immediately require the person to cease hoarding but, through an occupational perspective, support the individual to see value in engaging in differing activities to develop a position of occupational balance which better supports their health and wellbeing. It would seek to increase their occupational opportunities and occupational performance and could follow principles of harm reduction [89], enabling the person to move to a position of improved health as they feel ready to do so. Harm reduction approaches incorporate client-centered principles and engage clients in health promotion activities whilst ensuring minimum harm to the person through the treatment interventions [90]. Harm reduction approaches have been primarily used to treat substance abuse [91]; however, some studies have incorporated these approaches for hoarding [92, 93]. These studies propose the value of harm reduction interventions which would include collaboration with the individual and propose interventions other than forced decluttering. However, harm reduction approaches for hoarding also consider the level of risk to the individual from the excessive accumulated items in the home environment, and these include consideration of the environment, the individual themselves, and the level of support available. These aspects align well to the “Person-Environment and Occupation” (PEO) model [94] and thus could provide an opportunity to interlink an occupational therapy model with harm reduction approaches. Within this approach, an initial assessment by an occupational therapist would enable understanding of the risks of the environment on occupational engagement and occupational performance, the level of performance abilities the individual has, and their life skills to manage themselves and the home as well as the support available from others [95].

The principles of incorporating a client-centered approach with harm reduction principles to address hoarding include the acceptance of individual's choice and relate well to principles of occupational therapy in promoting an individual's engagement in personally meaningful activity [96] and in partnership and collaborative working [82]. These also correlate well with evidence that client-centered approaches lead to better outcomes when working with people who hoard [30, 53]. This leads to possibilities for occupational therapists to engage in work with people who hoard, to support them to find alternative health-sustaining occupations that are meaningful and chosen by them.

An understanding of hoarding, as a nonsanctioned occupation, provides opportunities for occupational therapists to appreciate the meaningfulness of hoarding to the individual, as well as acknowledging that hoarding may result in marginalisation because the activity does not conform to the predominant sociocultural expectations of a community [10]. It provides an opportunity for occupational science to inform occupational therapy practice to enable a better understanding of nonsanctioned occupations, such as hoarding, in order to perceive this as an occupation and not a medical disorder, which would then result in transformative client-centered approaches to support those who may have become marginalised because of their occupational choices [4].

These aspects can be related to recent health initiatives proposed by the Department of Health (2010) NHS England Five Year Forward View [97] and Public Health England [98], which aim to support populations to change their lifestyles and take initiatives to improve their health and wellbeing. Within these initiatives, it is acknowledged that allied health professionals can support people to improve their health and wellbeing, offering interventions which address physical and mental health issues [98, 99]. Thus, there is potential for occupational therapy to step forward into supporting those people who hoard to support them in enhancing their levels of occupational performance and engagement in order to improve their health and quality of life.

## 9. Conclusion

Literature pertaining to hoarding and hoarding activity, including previous research on the management of hoarding and use of legislative powers, has been explored. Such evidence has then been situated within the contemporary paradigm of the profession of occupational therapy in order to view hoarding as a daily activity and an occupation, which encompass roles and routines and provide purpose, self-identity, and personal meaning to an individual. Hoarding has been acknowledged as an activity which provides meaning and purpose to an individual but which also has an adverse impact on a person's health and wellbeing, with further recognition that there is a lack of acknowledgment in occupational therapy literature on how to consider nonsanctioned occupations, with hoarding being an example of this.

The author proposes that the opportunity to view hoarding from an occupational therapy and occupational science perspective can provide greater insight into aspects of hoarding as a meaningful purposeful activity. An understanding of hoarding as a daily occupation can also support consideration of alternative interventions to what are currently offered, appreciating that the removal of items, such as in a decluttering process, will not specifically stop a person hoarding if this is the person's daily occupation and constructs daily tasks, roles, and routines. Evidence shows high levels of recidivism after decluttering interventions and that the process of decluttering is actually harmful psychologically to the person, causing distress through the loss of cherished and meaningful items. The author proposes that this occurs because of a lack of acknowledgment for the meaning and value of engaging in hoarding activity and that without support to find alternative health sustaining activities the individual will revert to what is psychologically comforting and to what provides physical structure to their day, that is to say, hoarding.

Ensuring that an individual has purpose and opportunities to engage in meaningful occupations is a central tenant of occupational therapy, with the profession supporting concepts of occupational rights. However, whilst hoarding does provide meaningful purpose, roles, and routines, it is also evidenced as causing harm, physically and psychologically to an individual. There is therefore opportunity to support a person to engage in other meaningful activities and to develop alternative occupations which can replace hoarding, using approaches that incorporate occupational therapy values, principles, and approaches within a harm reduction approach. In addition to supporting an individual develop alternative activities, there is opportunity for occupational therapy interventions to address environmental restrictions in the home and the loss of functional skills which have occurred because of physical inabilities to access areas of the home such as the kitchen, supporting the individual to relearn functional skills which may have been lost.

Further research is needed to better understand hoarding as a daily occupation and to explore the potential for occupational therapists to work with people who hoard as an emerging area of practice.

## Figures and Tables

**Table 1:** 

Initial search terms	Hoarding; hoarding disorder, hoarding disorder, hoarding assessment, and hoarding treatment; hoarding and interventions; occupational therapy; occupational therapy and hoarding; hoarding and roles; hoarding and routines; health and wellbeing and hoarding; hoarding and occupational science
Revised search terms	Hoarding disorder and occupational therapy; hoarding disorder and treatment and assessment
	Specific parameters for assessment (risk/needs/symptom) were applied to narrow results. No parameters were applied to define treatment, but when reviewing titles of literature, treatment that was not peer-reviewed was excluded.
Inclusion criteria	Written in English
	Any literature which contained search terms of hoarding AND occupational therapy/occupational science
Exclusion criteria	Studies conducted on hoarding prior to 2000
	Hoarding studies conducted with children
	Hoarding studies focusing on specific types of hoarding, e.g., animal, shopping
	All duplicates found in other searches were removed.
